# Supplementary material for: Nanomechanical motion transduction with a scalable localized gap plasmon architecture
Source: Nat Commun. 2016 Dec 6;7:13746. doi: 10.1038/ncomms13746 (PMC5150643; doi:10.1038/ncomms13746)
Supplement: Supplementary Information — Supplementary Figures and Supplementary Notes [file ncomms13746-s1.pdf]

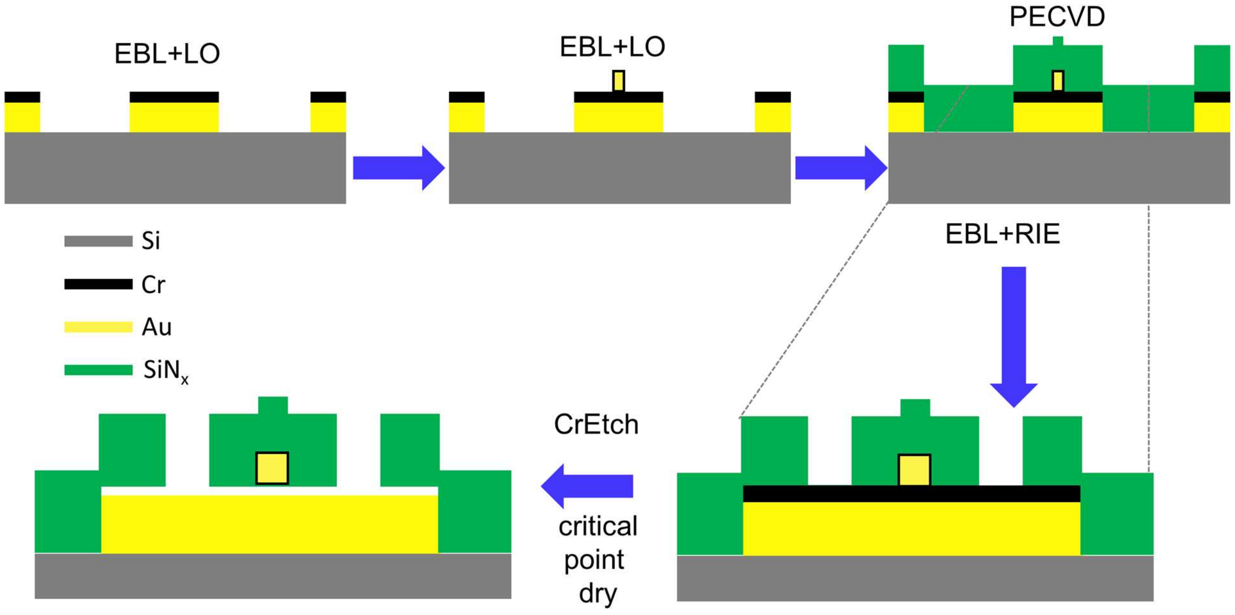

Supplementary Figure 1 | Fabrication process flow. Process flow for fabricating pNEMS. Processes are: EBL– electron beam lithography, LO – lift off, PECVD – plasma-enhanced chemical vapor deposition, RIE – reactive-ion etching.

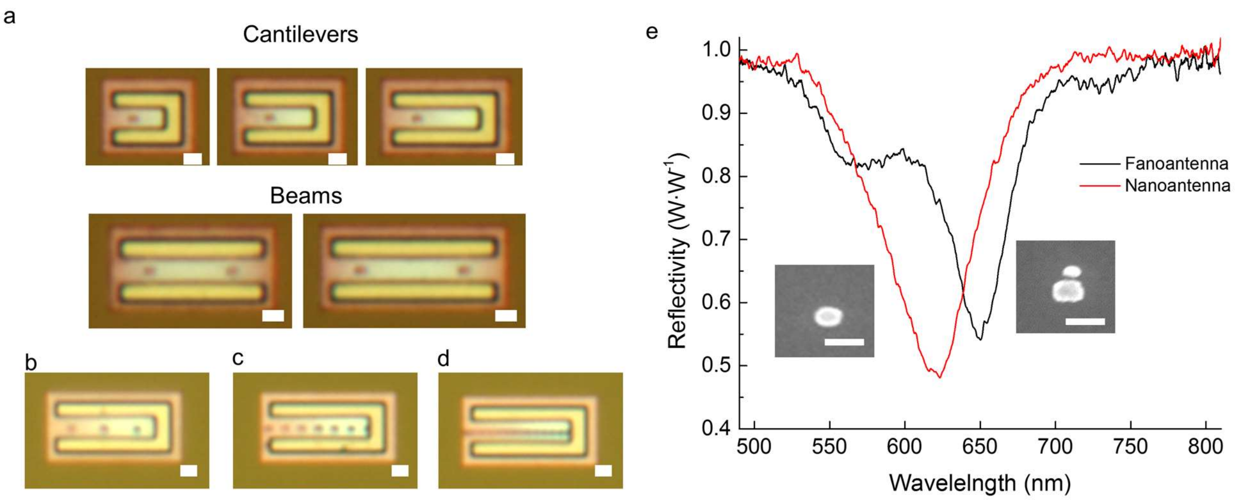

Supplementary Figure 2 | pNEMS design flexibility. (**a**) Variation of mechanical designs showing different length cantilevers and beams. (**b**)–(**d**), Variation of prism packing density. Prisms separated at (**b**) 2 μm (**c**) 1 μm, and (**d**) 0.5 μm are confined within functioning devices; scale bars (**a**)–(**d**) are 1 μm. (**e**) Measured reflectivity for different plasmonic resonators. The nanoantenna (left inset) has dimension 70×50 nm^2, whereas the fanoantenna comprises two nanorods (85×50 nm^2 and 45×25 nm^2) separated by a gap of approximately 10 nm; scale bars are 100 nm.

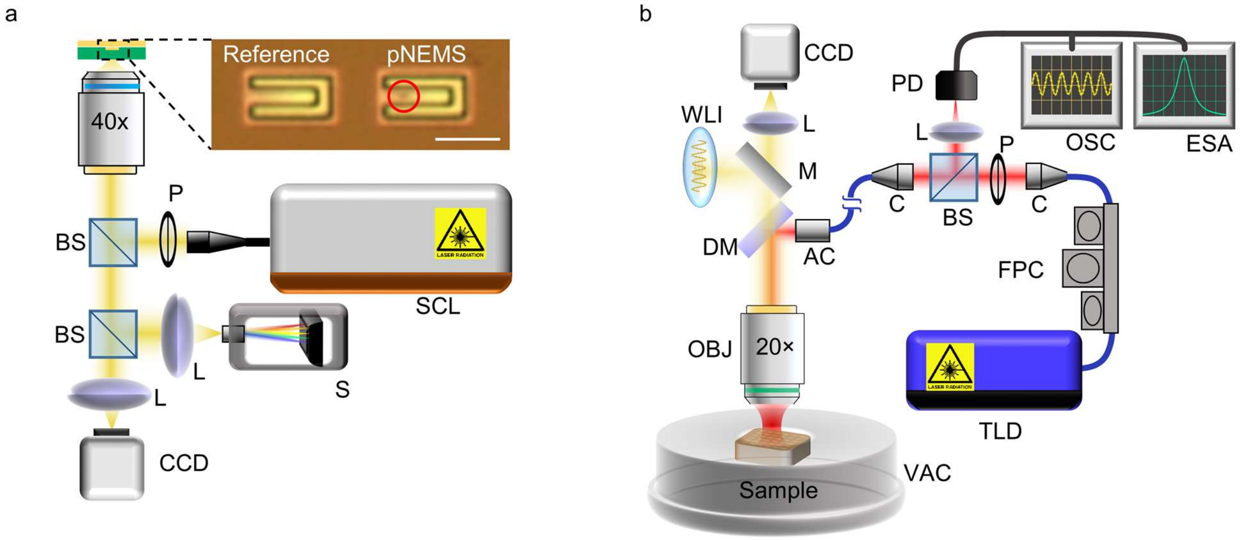

Supplementary Figure 3 | Experimental setups. (**a**) Schematic of the setup used for measurement of individual LGPs. A supercontinuum laser (SCL) is coupled into a 40×, 0.9 NA microscope objective through a 50-50 non-polarizing beamsplitter (BS); a polarizer (P) aligns the SCL polarization parallel to the prism long-axis. The reflected signal is split and simultaneously imaged onto a spectrometer (S) and a CCD using a 50:50 beamsplitter (BS) and tube lenses (L). The inset shows an optical image of a typical pNEMS device, prism location marked with a red circle, adjacent to a reference device; scale bar is 5 µm. (**b**) Schematic of the setup for motion measurement. A tunable fiber-coupled laser (TLD) is expanded with a collimator (C), passed through BS, and coupled into a polarization-maintaining fiber using C. Laser light from the fiber is expanded with an achromatic collimator (AC) and coupled into a microscope comprising a 20×, 0.3 NA objective, white-light illumination (WLI), dichroic mirror (DM), a 50% reflector (M) and CCD. The laser power is controlled using the fiber polarization controller (FPC) and P. Reflected signal from the sample inside the vacuum chamber (VAC) is coupled back into the optical train and reflected and focused onto a fast photodetector (PD) using BS and L. An oscilloscope (OSC) and spectrum analyzer (ESA) are used to monitor time and frequency domain signals, respectively.

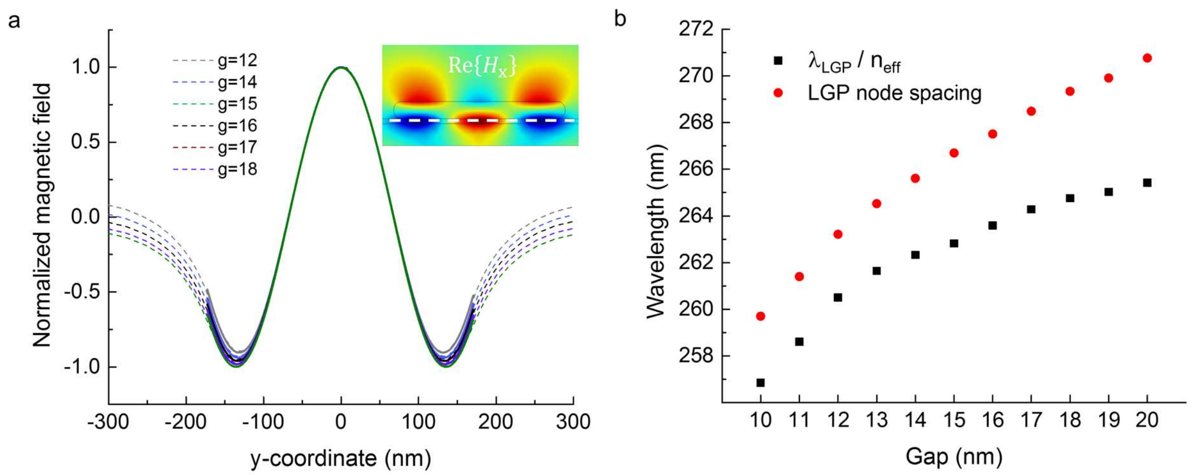

Supplementary Figure 4 | LGP mode validation. (**a**) Gap-dependent, normalized real component of the magnetic field calculated on a line through the center of the gap (dashed white line in inset), with sinusoidal fits (solid curves). (**b**) Comparison of resonant plasmon wavelengths in the resonator calculated from the combined 2D and 3D results (black squares) and taken directly from sinusoidal fits to 3D magnetic field distributions (red circles). Difference between the two curves is less than 2 %.

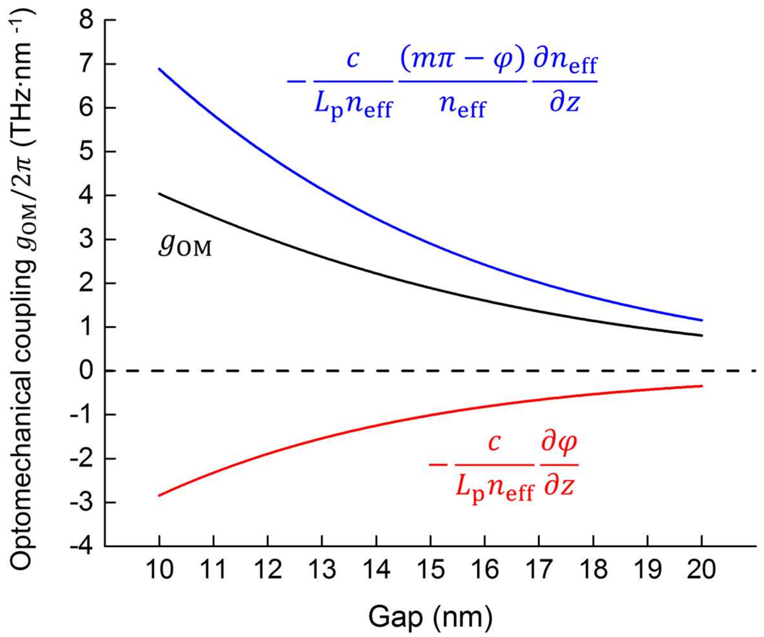

Supplementary Figure 5 | Optomechanical coupling contributions. Calculated values of g_{OM}(black curve) using the semi-analytical modal, alongside the effective index change term (blue curve) and the reflection phase change term (red curve).

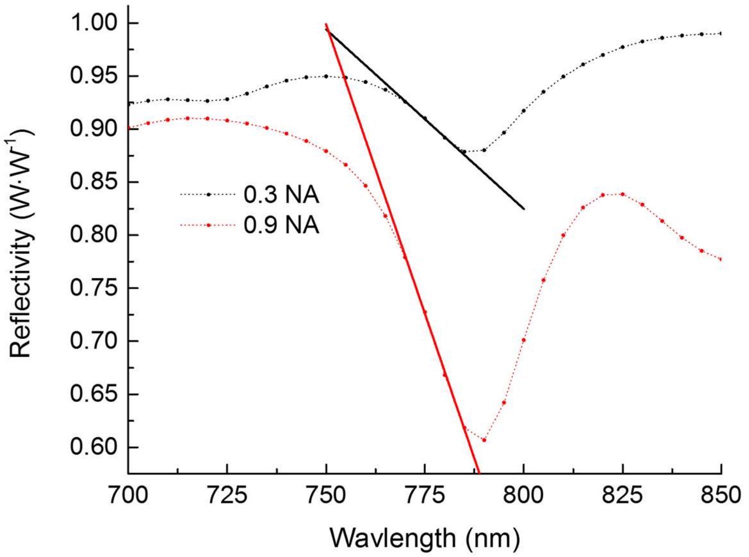

Supplementary Figure 6 | NA-dependent reflectivity. Comparison of the calculated reflectivity curves for 0.3 (black) and 0.9 (red) illumination. The curves are calculated for a 15 nm gap, and linear fits around 780 nm wavelength are included. The resonance visibility ratio is approximately equal to the ratio of illumination numerical apertures, as expected for a small resonator.

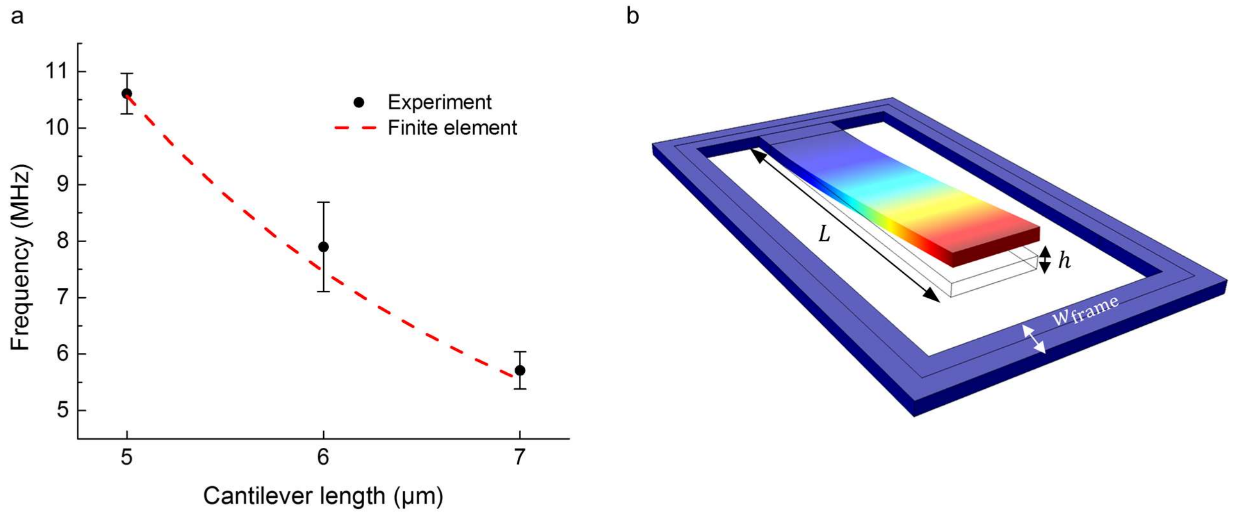

Supplementary Figure 7 | Cantilever frequency results and model. (**a**) Experimentally determined mechanical frequencies of the cantilever as a function of length with a finite element model. Error bars represent one s.d. for each cantilever device length, 10 measurements each. (**b**) Diagram of the mechanical model showing a cantilever and its supporting frame.

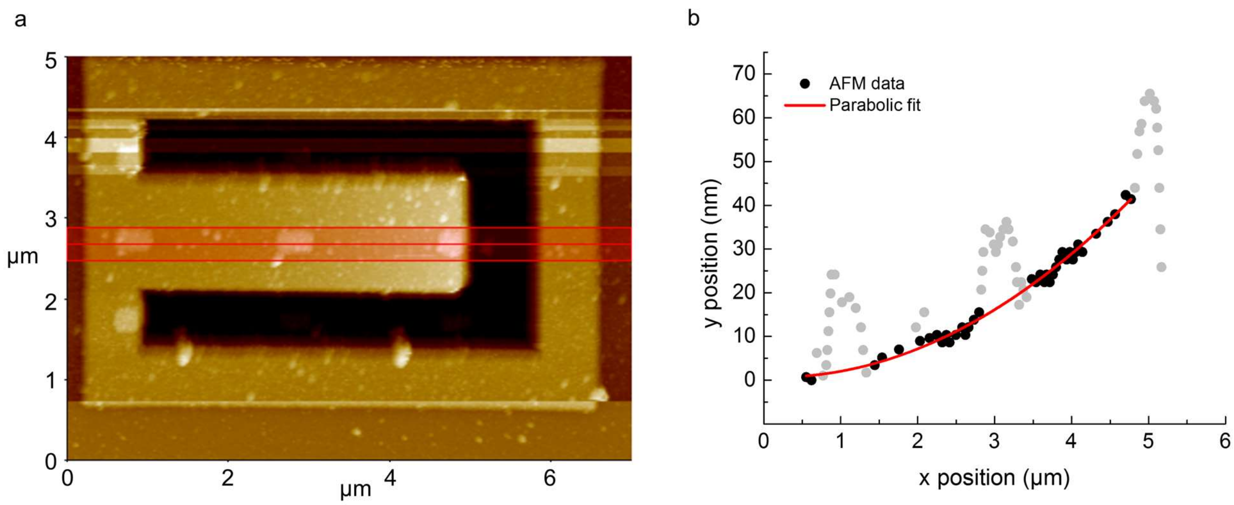

Supplementary Figure 8 | AFM results. (**a**) Two-dimensional topographic image of the test cantilever. (**b**) Height profile of measured through the center of the cantilever with a parabolic fit. Gray data points, indicating the height of the nitride cap above the embedded prisms, are not included in the fit.

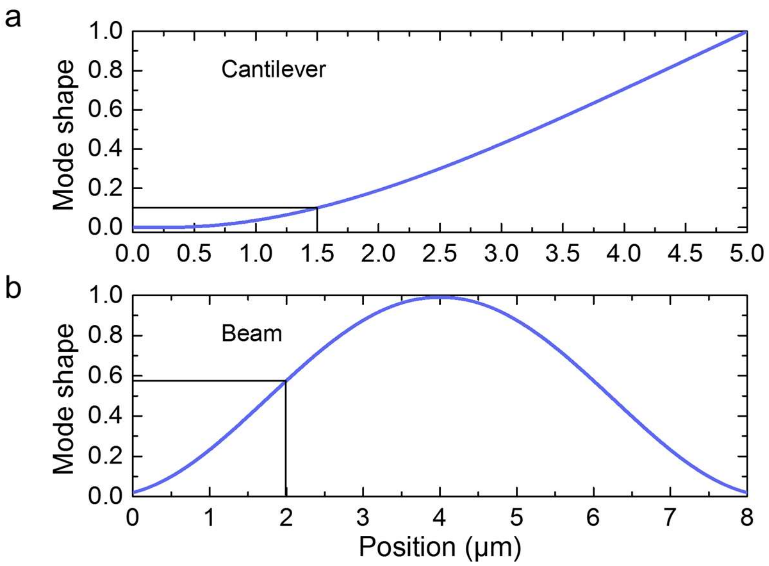

Supplementary Figure 9 | Normalized eigenmode shapes. Calculated mode shape for (**a**) the cantilever with $c_\mathrm{i} = 0.1$ and (**b**) the beam with $c_\mathrm{i} = 0.59$.

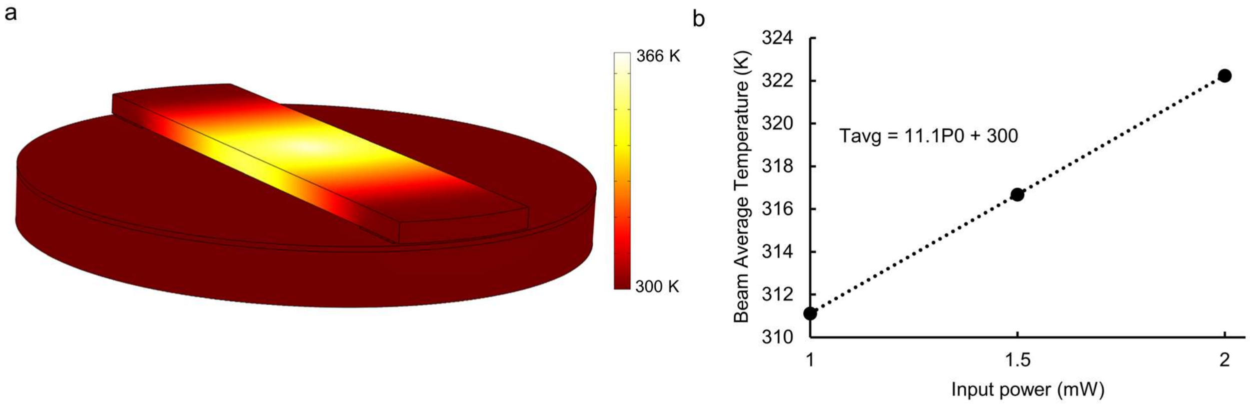

Supplementary Figure 10 | Plasmonic heating model. (**a**) Calculated temperature distribution in a SiN_x beam that is 1.25 μm wide, 5 μm long, and 175 nm thick. The prism is excited with a Gaussian beam focused to a diameter $w_0 = 1.2\lambda/\mathrm{NA}$, where λ = 780 nm, and NA = 0.3. (**b**) The average temperature calculated in the beam volume as a function of input power.

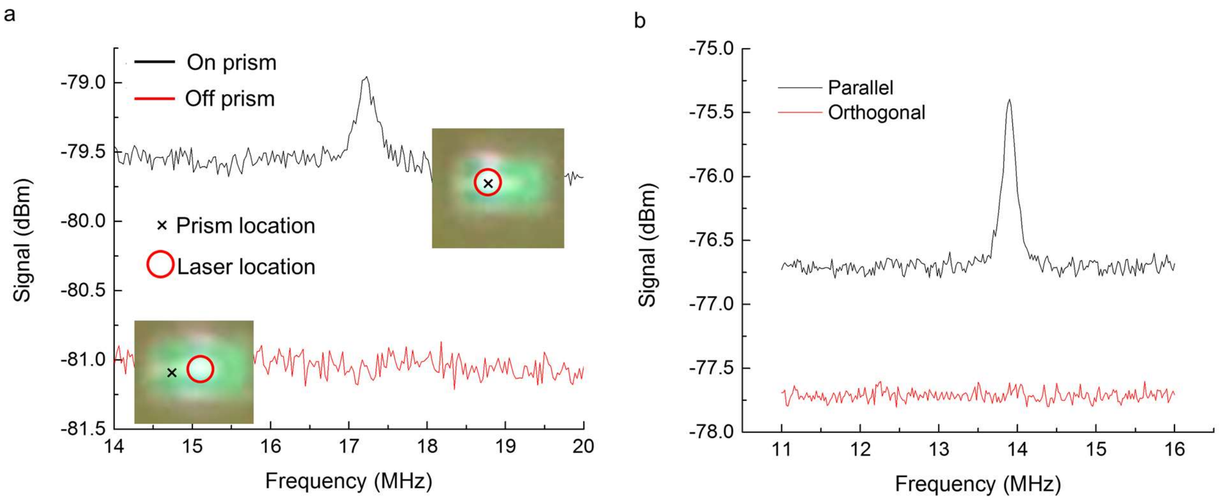

Supplementary Figure 11 | Control experiments. (**a**) Measured motion signals with a laser placed on the prism (black curve, top image) and off the prism (red curve, bottom image); prism position is marked with the × symbol, laser with a red circle. Dotted lines are drawn for reference with respect to the prism location. (**b**) Measured motion signal captured with TM polarization (black curve), which excites the desired plasmonic modes, and TE polarization (red curve).

Supplementary Note 1. Fabrication process for pNEMS

The fabrication process flow is given in Supplementary Fig. 1. The devices are built using repeated steps of aligned electron beam lithography (EBL) exposures. The first exposure uses a 495k/950k molecular weight polymethyl methacrylate bilayer resist (100 nm /30 nm thickness, respectively) and a base dosage of 1000 $\mu C \cdot cm^{-2}$ to define both chip alignment marks for subsequent layers and the pads upon which the mechanical resonators are built. After development in a mixture of methyl isobutyl ketone and isopropyl alcohol (1:3 by volume, respectively) for 60 s, electron beam evaporation is used to deposit a 3 nm Ti adhesion layer, a 45 nm Au pad, and a 12 nm Cr sacrificial layer. Critically, the Cr layer roughness is kept to a minimum by depositing with a high rate of 0.3 $nm \cdot s^{-1}$. This method prevents Cr adatom migration and clumping on the Au surface before a continuous film is established. Cr thickness is measured using a crystal balance within the chamber whose deposition rate is characterized with ellipsometry. After liftoff in n-methyl pyrrolidone, prisms are formed using EBL and the same bilayer resist with 2000 $\mu C \cdot cm^{-2}$ base dose, followed by 35 nm Au evaporation and liftoff. Here, the Cr sacrificial layer doubles as an adhesion layer for the prisms. The device layer is then formed using plasma-enhanced chemical vapor deposition (PECVD) to deposit a low-stress SiN_x layer at 180 °C with a nominal 175 nm thickness. Stress control represents a critical step in the fabrication process, as the residual stress and stress gradient cause the structures to deform away from the substrate. The low net residual compressive stress of approximately 150 MPa, as measured on a test wafer using a laser-interferometric stress analyzer, is achieved with radio frequency and inductively coupled plasma power of 50 W and 1100 W, respectively. A final EBL step is then used to form the beam etch mask composed of a 400 nm thick commercial high-resolution electron beam resist. After development for 60 s in room temperature hexyl acetate, the pattern is transferred to the nitride using reactive ion etching with a CF_4/CHF_3 (40 $cm^3 \cdot min^{-1}$/5 $cm^3 \cdot min^{-1}$) chemistry and 100 W radio-frequency power. After stripping the remaining resist using an O_2 plasma, devices are released using an extended (30 min) wet etch in a solution of ammonium ceric nitrate to completely remove the sacrificial layer and remaining Cr residue. This etchant is chosen due to its near complete chemical inertness to all materials in the process. Devices are finalized by an initial soak in isopropyl alcohol followed by critical point drying in liquid CO_2.

An advantage of the pNEMS architecture is the ability to tune the mechanical and plasmonic designs. Examples of mechanical design variation are shown in Supplementary Fig. 2a, wherein cantilevers and beams with varying lengths are produced. Similarly, the embedded plasmonic resonators can be varied in a straightforward manner. As shown in Supplementary

Figs. 2b–d, prisms can be placed at arbitrary distances within the NEMS with demonstrated separations from 2 μm (Supplementary Fig. 2b) down to 500 nm (Supplementary Fig. 2d). Variations in the resonator design are also possible. Supplementary Fig. 2e shows reflectivity measurements of released pNEMS containing varying types of nanoantenna design: 70×50 nm^2 nanorods and "fanoantennas" comprising two nanorods measuring 85×50 nm^2 and 45×25 nm^2 separated by a 10 nm gap. These data reflect two key points: (1) the operating spectral range of pNEMS can be simply tuned with resonator size and (2) initially designed plasmonic resonator shapes are retained within the released structures with high fidelity. The latter point is evidenced by the strong shoulder in the reflectivity for the fanoantenna, which occurs as a result of hybridization of the plasmonic modes between the narrowly separated nanorods.

Supplementary Note 2. Experimental setups

The first setup is used to probe LGP modes and consists of a broadband supercontinuum (SC) laser spanning a wavelength range of 500 nm to 2000 nm coupled into an inverted microscope with a 40×, 0.9 NA objective lens (Supplementary Fig. 3a). The SC laser is passed through an IR spectral filter to remove wavelengths greater than 850 nm, a neutral density filter to control intensity, a broadband polarizer to establish TM excitation of the sample, and a beam-expansion telescope to produce a 10 mm beam diameter that overfills the objective back aperture. Reflected light from the sample is imaged simultaneously onto a CCD for visual inspection and a fiber-coupled spectrometer for spectral analysis. In all cases, reported spectra are taken with 50 ms integration time and represent the average of 20 individual spectra. A reference spectrum (Ref) is first taken from a reference device without plasmonic structures by focusing the SC at 1.5 μm (2.0 μm) from the cantilever (beam) base. A closed-loop stage with positioning precision of less than 100 nm is used to move the device under test into the laser spot and a sample spectrum is taken (S). The LGP spectrum is then calculated by (S-D)/(Ref-D), where D is the detector dark spectrum collected with the laser off. For each prism measured, a new reference spectrum is collected in order to account for possible spectral drift in the SC source.

The second setup (Supplementary Fig. 3b) is used to measure mechanical responses and is based on a custom microscope assembly coupled to a vacuum chamber using a 20×, 0.3 NA glass-corrected, long-working distance objective. Laser light from a fiber-coupled diode laser is collimated to free space, passed through a beamsplitter, and then coupled into a polarization-maintaining, single-mode fiber. Light is delivered to the objective through an achromatic collimator, which produces a 7.5 mm output beam, and a dichroic mirror exhibiting 96% reflection over a 765 nm to 800 nm wavelength band. Optical power delivered to the

sample, measured using a photodetector inserted after the objective and before the vacuum chamber window, is set by a fiber-coupled polarization controller followed by a free-space polarizer. Reflected laser light is passed back through the same optical train and imaged on to 125 MHz bandwidth photodetector connected to an oscilloscope for measuring DC voltage. A fast-Fourier transform spectrum analyzer operating a 10 kHz resolution bandwidth is used to monitor the frequency domain signal. Reported spectra are the average of 500 individual scans taken over a 0.02 s window per scan. Measurements are performed with vacuum chamber pressure less than 0.1 Pa (10^{-3} Torr) to minimize frictional damping on the devices.

Supplementary Note 3. Electromagnetic calculations

For 3D electromagnetic calculations, a total-field formulation is used, in which a Gaussian beam (E_b) focused to a waist $1.2\lambda/\mathrm{NA}$ with NA = 0.9 is introduced into the system at the boundary. The domain comprises a Si substrate, 45 nm Au pad, embedded 350×165×35 nm^3 prism, and a 175 nm thick, 1.25 μm wide $\mathrm{SiN_x}$ slab suspended above the Au by a variable air gap. The $\mathrm{SiN_x}$ has a refractive index of 1.98 as determined from ellipsometry measurements of the deposited films; we use tabulated data for the optical constants of Au, and Si has a refractive index of 3.7. Perfectly matched layers surround the physical domains, and mirror symmetry is employed to reduce computational cost. The reflectivity is calculated as $|S_{11,\mathrm{samp}}|^2$ where $S_{11,\mathrm{samp}} = \iint (E_\mathrm{t} - E_\mathrm{b}) \cdot E_\mathrm{b} \mathrm{d}A \cdot \left(\iint E_\mathrm{b} \cdot E_\mathrm{b} \mathrm{d}A\right)^{-1}$ where integration is performed over the incident port area; $E_\mathrm{t} = E_\mathrm{b} + E_\mathrm{s}$ with scattered field E_s. As in the experiment, the device plasmonic far-field response is given by $|S_{11,\mathrm{samp}}|^2/|S_{11,\mathrm{ref}}|^2$, where $S_{11,\mathrm{ref}}$ is calculated without the prism.

The 2D eigenmode calculations are performed as function of gap size on a plane representing the center cross section of the 3D model, with the plane normal aligned with the prism long-axis. Eigenmode frequencies corresponding to the $\lambda_\mathrm{LGP}(z)$ values from the 3D model are used to determine the effective index and LGP wavevector of a fundamental gap plasmon mode travelling under a 165 nm wide slab of infinite extent along the prism length-axis. We also extract amplitude-normalized electric and magnetic field distributions for comparison with the 3D calculations. The field distributions of the 2D gap plasmon match the 3D distributions nearly identically, as shown in Figs. 2d–f in the main text. Furthermore, the 3D fields show distinct intensity extrema in the gap, whose period closely matches $\lambda_\mathrm{LGP}/n_\mathrm{eff}$, where n_eff is the gap plasmon effective index determined from the 2D eigenmode's wavevector. This correspondence is verified by fitting the real component of the x-polarized magnetic field (perpendicular to the prism long-axis) taken from a line in the middle of the prism-pad gap

(inset, Supplementary Fig. 4a) of the 3D numerical simulation to a sinusoidal function for the various gap sizes considered. The results of these fits are given in Supplementary Fig. 4a, which shows a close correspondence of fits to the numerical data. The period of these sinusoidal fits determine the standing-wave resonance plasmon wavelength of the LGP mode. Comparing the period with the physical wavelength of the LGP mode inside the gap, derived from the 2D eigenvalue numerical calculation $\lambda_{\mathrm{LGP}}/n_{\mathrm{eff}}$, we see that the two values agree to within 2 % for the parameter range investigated here (Supplementary Fig. 4b). These data show that the modes supported by the pNEMS are indeed standing wave gap plasmons.

The change in λ_{LGP} with increasing gap size is related to the optomechanical coupling constant via

$$g_{\mathrm{OM}} = \frac{\partial \omega_{\mathrm{LGP}}}{\partial z} = -\frac{2\pi c}{\lambda_{\mathrm{LGP}}^2}\frac{\partial \lambda_{\mathrm{LGP}}}{\partial z}, \tag{1}$$

where c is the speed of light, and ω_{LGP} is the angular frequency of the LGP mode. We therefore use the wavelength dependence $\partial\lambda_{\mathrm{LGP}}/\partial z$ to characterize the optomechanical coupling strength of the pNEMS. The semi-analytical expression for the optomechanical coupling constant, given in equation (2) of the main text, which combines the results of all computational data, consists of two terms relating to the change of effective index and reflected phase of the LGPs as the size of the gap changes. The relative contribution of the effective index and phase derivatives to the optomechanical coupling are shown in Supplementary Fig. 5. It can be seen that the contribution of the term dominated by the reflected phase derivative is deleterious to the overall coupling strength. Here, the negative value of g_{OM} indicates the blue shift in wavelength with increasing gap.

Supplementary Note 4. Motion transduction with reflectivity based measurement.

The motion transduction signal for our study is derived from the measurement of intensity reflected from the prism. The plasmonic response is modeled as a Lorentzian function

$$R(\lambda) = A_0 \frac{\Delta\lambda^2}{4(\lambda-\lambda_{\mathrm{LGP}})^2+\Delta\lambda^2}, \tag{2}$$

where A_0 is the amplitude, λ_{LGP} is the resonance wavelength of the localized gap plasmon, λ is the free-space wavelength, and the linewidth is given by $\Delta\lambda = \lambda_{\mathrm{LGP}}/Q_{\mathrm{LGP}}$, with quality factor Q_{LGP}. The plasmon resonance is probed by measuring the change in reflectivity,

$$\delta R = \partial R/\partial\lambda \cdot \frac{\partial \lambda_{\mathrm{LGP}}}{\partial z}\,\delta z, \tag{3}$$

thereby giving the reflectivity signal,

$$|\delta R| = \frac{8A_0\, Q_{\rm LGP}^2 \lambda_{\rm LGP}^2 (\lambda - \lambda_{\rm LGP})}{\left[4Q_{\rm LGP}^2(\lambda-\lambda_{\rm LGP})^2 + \lambda_{\rm LGP}^2\right]^2} \cdot \frac{\partial \lambda_{\rm LGP}}{\partial z}\, \delta z \,. \tag{4}$$

The reflectivity signal is a maximum at $\partial^2 R/\partial\lambda^2 = 0$, which occurs at wavelengths

$$\lambda_{\rm s} = \lambda_{\rm LGP}\left(1 \pm \frac{1}{2\sqrt{3}\, Q_{\rm LGP}}\right), \tag{5}$$

on the blue (-) and red (+) side of the resonance. Probing the reflectivity at $\lambda_{\rm s}$ gives

$$\delta R(\lambda_{\rm s}) = \frac{3\sqrt{3}\, A_0\, Q_{\rm LGP}}{4\, \lambda_{\rm LGP}} \cdot \frac{\partial \lambda_{\rm LGP}}{\partial z}\, \delta z \,, \tag{6}$$

therefore

$$\frac{\delta R}{\delta z}(\lambda_{\rm s}) \propto Q_{\rm LGP} g_{\rm OM}. \tag{7}$$

The optomechanical coupling constant for the pNEMS is determined using

$$g_{\rm OM}/2\pi \equiv \frac{1}{2\pi}\frac{\partial \omega_{\rm LGP}}{\partial z} = \frac{c}{\lambda_{\rm p}^2}\eta \left|\frac{1}{R_0}\frac{\partial R}{\partial \lambda}\right|^{-1} \frac{\alpha}{P_0\, G_{\rm DC}}, \tag{8}$$

where $\lambda_{\rm p}$ is the wavelength used to probe the mechanical resonance, P_0 is the power measured at the photodetector (45 μW), R_0 is the reflectivity at $\lambda_{\rm p}$, and $G_{\rm DC}$ is the photodetector gain measured to be 25 mV·μW^{-1} for one mW of input optical power. In order to account for the changes in slope $\partial R/\partial\lambda$ expected from use of different measurement setups, we include the factor $\eta \approx 3.3$. This value accounts for the reduction in slope for mechanical measurements, performed with a 0.3 NA objective, compared to spectroscopic measurements performed at 0.9 NA. The numerically determined factor is approximately equal to the ratio of the NA of the two systems, as would be expected in the limit of small optical cross section (Supplementary Fig. 6).

Supplementary Note 5. Mechanical model and calibration

5.1 Determination of device clamping conditions and stress state

In order to determine the mechanical behavior of the pNEMS, we combine the results of finite-element calculations with mechanical frequency ($\Omega_{\rm m} = 2\pi f_{\rm m}$) measurements of a series of test devices. The resonant frequency of the devices can be expressed as

$$\Omega_{\rm m} = \frac{A}{\sqrt{12}}\frac{h}{L^2}\sqrt{\frac{E}{\rho}}\,, \tag{9}$$

for a nitride beam with length L, a rectangular cross section of thickness h, elastic modulus E, and density ρ. Here, A is a parameter that represents the clamping conditions of the device with values of 3.52 and 22.4 for an ideal singly and doubly-clamped beam rigidly attached at their

bases, respectively. Using a commercial Doppler vibrometer system, we measure f_{m} for a set of nominally 5 μm, 6 μm, and 7 μm cantilevers by focusing the 633 nm wavelength laser at the free end of the devices where thermally excited vibration amplitude is largest. Cantilevers are chosen specifically for this characterization due to the fact that the initial film stress does not significantly affect their f_{m} values due to stress relaxation of the devices upon release.

Supplementary Fig. 7a shows the results of the measurements along with the values of f_{m} predicted from a finite element mechanical model (broken red curve). The model is based on the geometry shown in Supplementary Fig. 7b, in which the cantilever or beam is attached to a frame with a designed width w_{frame} of 500 nm. In order to match the finite-element predicted frequencies with experimental values, the material parameters, clamping conditions (equivalently A), and device dimensions must be known. The dimensions are taken from SEM images of the devices giving thickness of 185 nm and 165 nm for the 5 μm cantilever and 8 μm beam, respectively. For material parameters, we use an elastic modulus of 220 GPa (determined from nanoindentor measurements), a Poisson ratio of 0.2, and a mass density of 2200 kg·m^{-3}.

We find that for devices with the outer half of the frame rigidly attached at the bottom (a bottom clamp width of $w_{\mathrm{clamp}}/2$), good agreement is obtained between the measured and predicted f_{m} values. We therefore assume all devices are clamped in this manner, giving A= 3.97 for the cantilever. However, a 5 μm cantilever with a measured frequency f_{m} = 13.5 MHz was used for the measurements reported in the main text. This frequency is at the higher end of the frequency distribution for nominally identical cantilevers. Inasmuch as the material parameters of the SiN_x are not likely to vary significantly between physically adjacent devices, we explain the frequency distribution by the variation of the width of the bottom clamping region, which is tentatively attributed to defects in the Cr sacrificial layer near the edge of the bottom Au pad. Based on this frequency, this cantilever, in addition to the "baseline" clamping, is rigidly attached a distance 350 nm along its length from its attachment to the frame. Since all the cantilevers are curling up slightly from their bases due to the residual stress gradient (see below), this cantilever was chosen because it has the smallest additional increase in the gap from its nominal value, and therefore expected to have the highest optomechanical coupling.

Contrary to cantilevers, the resonance frequency in the beams is expected to be a function of the residual stress, and is expected to be reduced by the compressive stress of the fabricated SiN_x. The measured f_{m} for the 8 μm beam in the main text (approximately 15 MHz) is significantly lower than the predicted value from the zero-stress baseline model. Indeed, we

find that $A = 11.6$ for this device. To account for this fact, we, again, use an FEM model of the structure clamped on the outside of the frame and apply an initial residual stress state of $\sigma(z) = \sigma_0 + 2|\sigma_0|/h\,z$, where σ_0 = -180 MPa, which closely reproduces experimentally observed f_{m}. The applied residual stress value is in good agreement with the measured value of -150 MPa using a wafer-bow technique.

As stated in the main text and Supplementary Note 1, the residual stress gradient is responsible for the cantilever curling away from the substrate after release (facilitating the release process). To understand the magnitude of this deformation, and thus its ultimate impact the LGP resonator gap, resonant wavelength, and optomechanical coupling constant, we perform atomic force microscopy (AFM) measurements on a 4 μm test cantilever (Supplementary Fig. 8a). Here, a shorter cantilever is used to provide a higher overall stiffness to facilitate the AFM measurement, however, the radius of curvature originating from the stress gradient is not significantly affected by the shorter length.

Fitting the AFM height data to a parabola (Supplementary Fig. 8b) gives the curvature of the nitride due to the stress gradient. Using this curvature, we find that the experimentally determined maximum deflection value at the tip of a 5 μm cantilever is 60 nm, which agrees well with theoretically predicted value of 75 nm under the assumption of the linear stress gradient stated above. From these data, we predict that the maximum gap size for an LGP resonator 1.5 μm from the base, comprising the sum of 12 nm sacrificial layer and stress-induced deflection, is 16 nm. Similarly, for the 8 μm beam, we find a theoretically predicted beam deflection away from the substrate of 15 nm at the beam center, resulting in a net 21 nm gap at the prism location. A ± 1 nm error bar in the gap size determined from this method corresponds to the s.e. of the parabolic fit.

5.2 Calculation of modal mass, modal displacement, and effective stiffness.

Using the finite element model above, we determine the modal mass by extracting the normalized mode shape Φ for the fundamental mode of the device, which gives $m_{\mathrm{eff},q} = \int \rho |\Phi|^2 \mathrm{d}V / q^2$ where q a generalized coordinate for the prism displacement. The clamping conditions found above play an important role in determining the ratio of the movement of the device at the location of the prism to the maximum modal displacement, denoted c_{i}. The c_{i} values, which determine the effective stiffness of the device mechanical mode as seen by the LGP resonator via $\kappa_{\mathrm{eff}} = m_{\mathrm{eff}} \Omega_{\mathrm{m}}^2 \cdot c_{\mathrm{i}}^{-2}$, are extracted from the mode shapes as shown in Supplementary Fig. 9.

5.3 Plasmonic heating effects

Absorption of the input probe laser by the LGP resonator results in a temperature increase of the system, which thereby affects the thermal motion of the system. To account for this, we calculate the temperature distribution of a 5 μm long, 1.25 μm wide, 175 nm thick nitride beam with an embedded prism and a 15 nm gap, excited by an incident 0.3 NA Gaussian beam with 1.7 mW input power. One quarter of the geometry is simulated using symmetry conditions to reduce the computation cost, and the model parameters are chosen to match the experimental conditions for the motion measurement with a 2 mW input power, of which 85 % actually reaches the sample through the uncoated glass window on the vacuum chamber. The beam is connected to a 45 nm thick Au pad atop a 500 nm thick Si substrate by a 500 nm × 1250 nm rectangular support serving as a thermal contact between the beam and substrate. All boundaries are set as thermal insulation with exception of the bottom of the Si substrate, which is set to T = 300 K. Supplementary Fig. 10a shows the resulting temperature distribution of the pNEMS device, whereas Supplementary Fig. 10b shows the calculated average temperature of the beam.

Supplementary Note 6. Control Experiments

To confirm that the motion transduction signals are indeed due to the LGPs, we perform two control experiments detailed in Supplementary Fig. 11. All devices show similar behavior in these experiments, and we present data from two randomly selected cantilevers (one for each control experiment) from the same array as the device described in the main text. In the first experiment, the excitation laser is first placed directly over the plasmonic prism inside a cantilever, and then subsequently moved along the cantilever length by 2 μm to a position without a prism. The measured motion signal (black curve, Supplementary Fig. 11a) disappears completely once the laser is moved (red curve, Supplementary Fig. 11a), thereby showing that the prism is required to transduce the pNEMS motion. The slight reduction in signal power for the off-prism data is caused by non-perfectly parallel translation of the probe laser with respect to the cantilever long axis, which reduced slightly the overall reflected optical power that is confocally collected. In the second experiment, the polarization of the excitation source is rotated from a direction parallel to the prism length (Supplementary Fig. 11b, black curve) to the orthogonal direction (Supplementary Fig. 11b, red curve). Elimination of the signal due to polarization rotation is strong evidence of the plasmonic nature of the motion transduction. In this case, the slightly lower signal power for the orthogonal polarization is caused by systematic reductions in the power delivered to the sample through our optical system. We nevertheless observe no motion transduction for this input polarization upon increasing the input power up to

the instrumental limits of our system (3 mW). Notwithstanding these experimental subtleties, the observed behavior, i.e., elimination of the motion signal, is consistent with the data presented in Supplementary Fig. 11 for all devices tested in this study.
